# Dual inhibition of EGFR at protein and activity level via combinatorial blocking of PI4KIIα as anti-tumor strategy

**DOI:** 10.1007/s13238-014-0055-y

**Published:** 2014-05-07

**Authors:** Jiangmei Li, Lunfeng Zhang, Zhen Gao, Hua Kang, Guohua Rong, Xu Zhang, Chang Chen

**Affiliations:** 1 National Laboratory of Biomacromolecules, Institute of Biophysics, Chinese Academy of Sciences, Beijing, 100101 China; 2 University of Chinese Academy of Sciences, Beijing, 100049 China; 3 Department of General Surgery, Xuanwu Hospital, Capital Medical University, Beijing, 100053 China; 4 Tianjin Research Center of Basic Medical Science, Tianjin Medical University, Tianjin, 300070 China; 5 Beijing Institute for Brain Disorders, Beijing, 100069 China

**Keywords:** phosphatidylinositol 4-kinase IIα (PI4KIIα), EGFR, dual inhibition, enhanced anti-tumor effect, breast cancer, Iressa

## Abstract

Our previous studies indicate that phosphatidylinositol 4-kinase IIα can promote the growth of multi-malignant tumors via HER-2/PI3K and MAPK pathways. However, the molecular mechanisms of this pathway and its potential for clinical application remain unknown. In this study, we found that PI4KIIα could be an ideal combinatorial target for EGFR treatment via regulating EGFR degradation. Results showed that PI4KIIα knockdown reduced EGFR protein level, and the expression of PI4KIIα shows a strong correlation with EGFR in human breast cancer tissues (*r* = 0.77, *P* < 0.01). PI4KIIα knockdown greatly prolonged the effects and decreased the effective dosage of AG-1478, a specific inhibitor of EGFR. In addition, it significantly enhanced AG1478-induced inhibition of tumor cell survival and strengthened the effect of the EGFR-targeting anti-cancer drug Iressa in xenograft tumor models. Mechanistically, we found that PI4KIIα suppression increased EGFR ligand-independent degradation. Quantitative proteomic analysis by stable isotope labeling with amino acids in cell culture (SILAC) and LC-MS/MS suggested that HSP90 mediated the effect of PI4KIIα on EGFR. Furthermore, we found that combined inhibition of PI4KIIα and EGFR suppressed both PI3K/AKT and MAPK/ERK pathways, and resulted in downregulation of multiple oncogenes like PRDX2, FASN, MTA2, ultimately leading to suppression of tumor growth. Therefore, we conclude that combined inhibition of PI4KIIα and EGFR exerts a multiple anti-tumor effect. Dual inhibition of EGFR at protein and activity level via combinatorial blocking of PI4KIIα presents a novel strategy to combat EGFR-dependent tumors.

**Electronic supplementary material:**

The online version of this article (doi:10.1007/s13238-014-0055-y) contains supplementary material, which is available to authorized users.

## Introduction

Phosphatidylinositol 4 kinase IIα (PI4KIIα) is the dominant PI4K in mammalian cells. It is mainly localized with the *trans*-Golgi network and endosomes (Lu et al., 2012; Minogue and Waugh, 2012). The results of our previous study indicated that PI4KIIα is a novel regulator of tumor-related processes, including angiogenesis and HIF-1α accumulation, by way of activating HER-2/PI3K and ERK cascades (Li et al., 2010). Other studies showed that PI4KIIα is involved in pathways related to tumor progression, for example, Pan et al. (Pan et al., 2008) and Qin et al. (Qin et al., 2009) reported that PI4KIIα is important for WNT signaling pathway; Chu et al., (2010) reported that PI4KIIα subtype specifically influences AKT activity. Christina et al. reported that inositol polyphosphate 4-phosphatase type II (INPP4B) is a tumor suppressor (Gewinner et al., 2009), indicating that the D-4 position phosphorylated productions of phosphatidylinositol (PI) are essential for tumor growth. All above suggest that PI4KIIα could be a promising target for cancer therapy. However, the application significance and molecular mechanisms behind these links remain unclear.

Previous reports have established the relationship between PI4KIIα and ERBB families: Scott et al., (1991) showed that PI4K activity increases after stimulation of the ERBB-2 (HER-2). Kauffmann-Zeh et al., (1994) reported that EGF regulates PI4KIIα activity and is associated with ERBB-1 (EGFR), and Minogue et al., (2006) demonstrated that PI4KIIα influences the endosomal trafficking of EGFR. Our own work demonstrated previously that knockdown of PI4KIIα drastically reduces HER-2 autophosphorylation (Li et al., 2010). The activation of ERBB families is involved in controlling diverse cellular responses such as proliferation, differentiation, motility, and apoptosis as well as tumorigenesis (Flynn et al., 2009; Harandi et al., 2009; Tai et al., 2010).

Here we examined the regulatory mechanisms by which PI4KIIα controls EGFR protein levels, and tested the effect of dual inhibition of PI4KIIα and EGFR on tumor growth. We found that EGFR inhibition at both protein and activity level via combinatorial blocking of PI4KIIα proved superior as a strategy to suppress EGFR-dependent tumor growth. The combined anti-tumor mechanisms were also investigated.

## Results

### Regulation of HER-2 activity and EGFR protein levels by PI4KIIα

**Figure 1:**
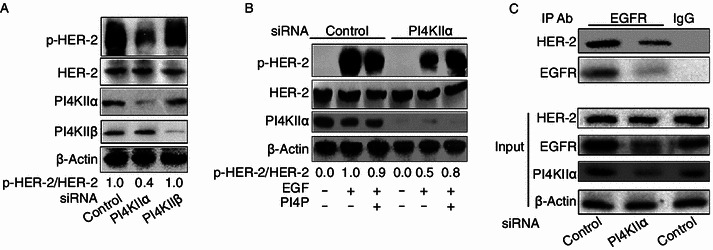
Effect of PI4KIIα knockdown on both HER-2 activity and HER-2/EGFR interaction. (A) Specific regulation of HER-2 by PI4KIIα. MCF-7 cells were transfected with either control siRNA, PI4KIIα siRNA or PI4KIIβ siRNA, and treated with 100 ng/mL EGF for 10 min. The autophosphorylation level of HER-2 at Tyr1248 and the protein expression levels as indicated were measured by Western blot. (B) The rescue effect of PI4P on HER-2 activity following PI4KIIα knockdown. (C) Effect of PI4KIIα knockdown on the interaction of HER-2 with EGFR. Lysates of treated MCF-7 cells (prior to addition of EGF for 10 min) were subjected to immunoprecipitation with anti-EGFR antibody or rabbit IgG respectively. The immunoprecipitates were immunoblotted with anti-HER-2 antibody and EGFR antibody. Loading controls are shown in the lower panels (Input). All results presented above represent data from three independent experiments

We have previously reported that the reduction in PI4KIIα level diminishes the autophosphorylation activity of HER-2, whilst its protein level remains unchanged (Li et al., 2010). Here we further indicated that the suppression of PI4KIIα, but not PI4KIIβ, resulted in downregulation of HER-2 activity (Fig. 1A), and this effect was reversed by direct delivery of phosphatidylinositol 4-phosphate (PI4P) (Fig. 1B). Above results suggest this regulation is PI4KIIα subtype-specific and kinase activity dependent. Since PI4KIIα RNAi does not affect HER-2 expression, we wondered whether the effect of PI4KIIα on HER-2 activity instead interferes with the formation of HER-2/EGFR heterodimers, and the result shown in Fig. 1C confirmed this suspicion. At the same time, we noted that the protein level of EGFR was much lower in PI4KIIα RNAi cells (Fig. 1C).

**Figure 2:**
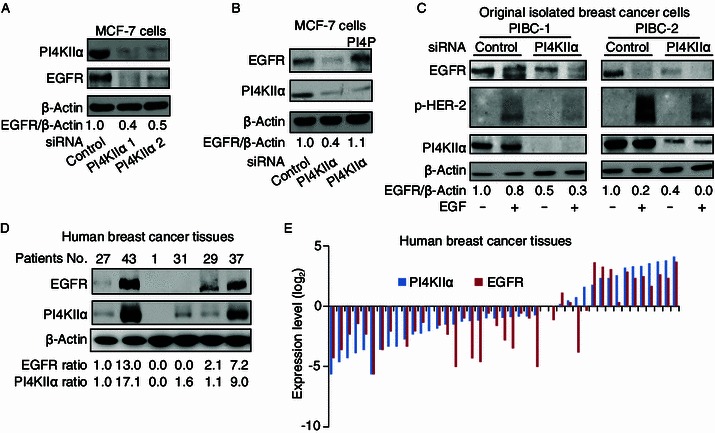
Regulation of EGFR protein levels by PI4KIIα knockdown. (A) Effect of PI4KIIα inhibition on EGFR protein levels. MCF-7 cells were transfected with either control siRNA or PI4KIIα siRNA (PI4KIIα 1 and PI4KIIα 2 represent two siRNAs targeting different sites of the PI4KIIα mRNA), and indicated proteins measured by Western blot. (B) The rescue effect of PI4P on EGFR levels following PI4KIIα knockdown. (C) Effect of PI4KIIα knockdown on EGFR protein levels in primary isolated breast cancer cells. PIBC1 and PIBC2, were transfected with control or PI4KIIα siRNA, and then treated with EGF for 10 min, prior to Western blotting for proteins indicated. All results presented above represent data from three independent experiments. (D) Representative examples of PI4KIIα and EGFR expression in breast cancer tissues detected by immunoblotting. (E) Statistical analysis of PI4KIIα and EGFR expression in breast cancer tissues. Protein log_2_ fold changes (on the y axis) are compared to the protein level of No. 27 patient

To confirm the regulation of EGFR by PI4KIIα, we tested this effect in different cell lines: MCF-7 cells (human breast adenocarcinoma cell line, Fig. 2A), MDA-MB-231 cells (triple-negative breast cancer cell line, Fig. S1A) and A549 cells (non-small-cell lung carcinoma (NSCLC) cell line, Fig. S1B), and all results confirmed that PI4KIIα knockdown resulted in EGFR reduction. In addition, PI4P could completely rescue the reduction of EGFR protein level induced by PI4KIIα knockdown (Fig. 2B). We further examined the effect of PI4KIIα on EGFR in two different primary isolated breast cancer cells (PIBC-1 and PIBC-2). As shown in Fig. 2C, the regulatory connection between PI4KIIα and EGFR/p-HER-2 is also present in primary isolated breast cancer cells. Then we detected the expression of both proteins in 43 human breast cancer tissues, Fig. 2D shows the immunoblotting results of the expression of EGFR and PI4KIIα in representative human breast cancer samples and Fig. 2E is a statistical analysis of all tested samples. The result of Pearson correlation coefficient analysis indicated that a strong correlation of EGFR and PI4KIIα expression existed in these breast cancer patients (*r* = 0.77, *P* < 0.01). To investigate if the effect of PI4KIIα on EGFR protein level is dependent on HER-2 activity, we examined it in the HER-2 negative MDA-MB-231 cells. The results indicate that this effect is independent of HER-2 (Fig. S1A).

### Enhanced effects of PI4KIIα knockdown and EGFR inhibitor action on EGFR function

**Figure 3:**
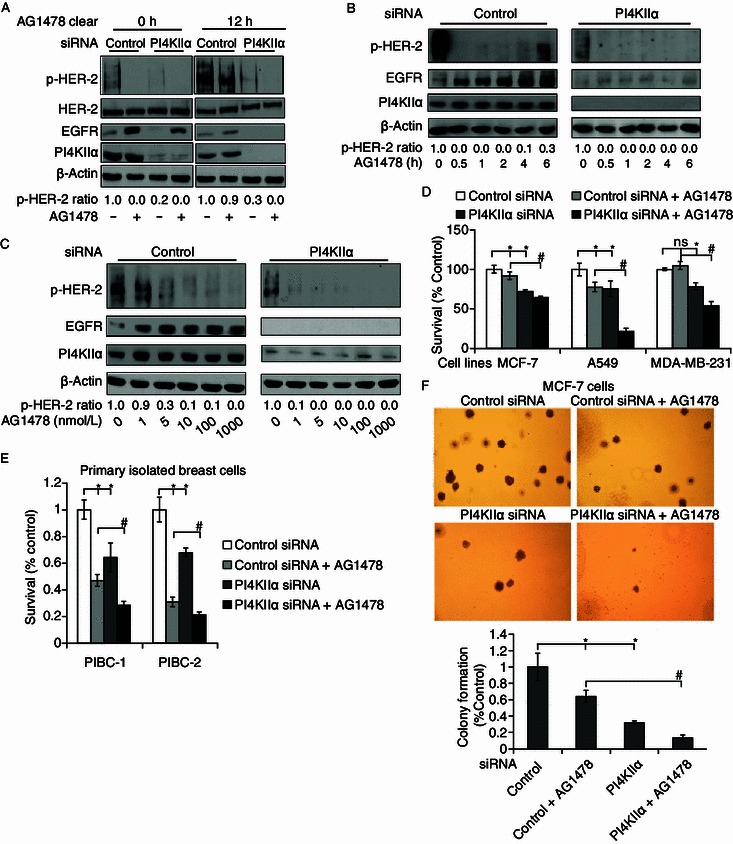
Ehanced effects of PI4KIIα knockdown and EGFR inhibition by AG1478 on HER-2 activity and cell viability. (A–C) Time and dose effect of AG1478 on p-HER-2 in PI4KIIα siRNA-treated cells and controls. MCF-7 cells were transfected with control or PI4KIIα siRNA before the following treatments (A–C), EGF was added for 10 min, lysed the cells and analyzed the levels of phosphorylated HER-2, the expression levels of EGFR and PI4KIIα by Western blot, with β-actin as the loading control. (A) 100 nmol/L AG1478 was added into the cell medium for 2 h except for controls, and then removed by washing cells for three times using PBS, cells were lysed or cultured as indicated for another 12 h. (B) Cells were incubated with 100 nmol/L AG1478 for indicated times. (C) Cells were treated with different concentrations of AG1478 for 2 h. (D) Effect of PI4KIIα knockdown and AG1478 (5 μmol/L) on cell viability of MCF-7 cells, A549 cells and MDA-MB-231 cells. (E) Effect of PI4KIIα suppression and AG1478 on cell viability of two primary isolated breast cancer cells. Primary cell lines PIBC1 and PIBC2 were transfected with control or PI4KIIα siRNA for 24 h, and then treated with 5 μmol/L AG1478 for another 48 h, except controls. (F) Effect of PI4KIIα suppression and AG1478 on colony formation in MCF-7 cells. Anchorage-independent cell growth was measured using a soft agar assay. Colony numbers were determined after 14 days incubation in soft agar. Data are presented as mean ± SD, *P < 0.01, as compared to control RNAi cells, while ^#^*P* < 0.01, as compared to cells treated with both AG1478 and control RNAi. All results presented above represent data from three independent experiments

Based on the observation that PI4KIIα knockdown reduced EGFR protein levels, we asked whether dual control of protein level and activity of EGFR results in enhanced effects during anti-cancer therapy. To this end, we first investigated whether AG1478 (a specific EGFR inhibitor) is more efficient in PI4KIIα suppressed cells. As shown in Fig. 3A, both AG1478 and PI4KIIα knockdown significantly suppressed HER-2 activity. Interestingly, even though AG1478 is removed from the cell culture medium after 12 h, its inhibitory effect is retained in MCF-7 cells treated with PI4KIIα RNAi. In contrast, the inhibitory effects completely disappeared in the control samples. We then treated cells with a combination of PI4KIIα siRNA and 100 nmol/L AG1478 for different times, and found that the effect of AG1478 was weakened after 6 h of incubation in the control cells, but its effect remained the same in PI4KIIα RNAi cells (Fig. 3B). All above results indicate that PI4KIIα knockdown has the ability to extend the effective time of AG1478. Then we determined the effects of PI4KIIα suppression on AG1478 effective dosage, and as shown in Fig. 3C, the effective dose of AG1478 was about 5 nmol/L in control cells, while just 1 nmol/L of AG1478 was sufficient to exhibit significant effects in PI4KIIα RNAi cells.

Next, we investigated the combinatorial effect on cell viability. As shown in Fig. 3D, combined inhibition of PI4KIIα and EGFR was more effective in inhibiting cell viability compared to single application of either method in the three cell lines as indicated, in particular in NSCLC A549 cells and the triple-negative breast cancer cell line MDA-MB231 cells. Singular treatment with 5 μmol/L AG1478 reduced the number of viable A549 control cells by approximately 22.5%, but by more than 78% in combinational treated A549 cells. Addition of 5 μmol/L AG1478 resulted in approximately 50% inhibition of cell proliferation in PI4KIIα RNAi MDA-MB-231 cells, but showed no inhibitory effects at all in the control MDA-MB-231 cells. The enhanced effects were confirmed in primary isolated breast cancer cells (Fig. 3E), indicating that the observed effects are not limited to cell lines. Using a soft agar assay, we measured the enhanced effects of PI4KIIα knockdown and EGFR inhibitor on anchorage-independent growth of MCF-7 cells (Fig. 3F). 10 μmol/L AG 1478 yielded a reduction in cell colony formation by approximately 36%, and PI4KIIα knockdown reduced the number of colonies by approximately 67%. In contrast, combinatorial treatment achieved an 87% reduction in colony formation. Together, these results indicate that combined treatment with PI4KIIα siRNA could significantly enhance the AG1478 anti-tumor effects in cell level.

### Combinatorial effects of PI4KIIα and EGFR on suppression of tumor growth *in vivo*

**Figure 4:**
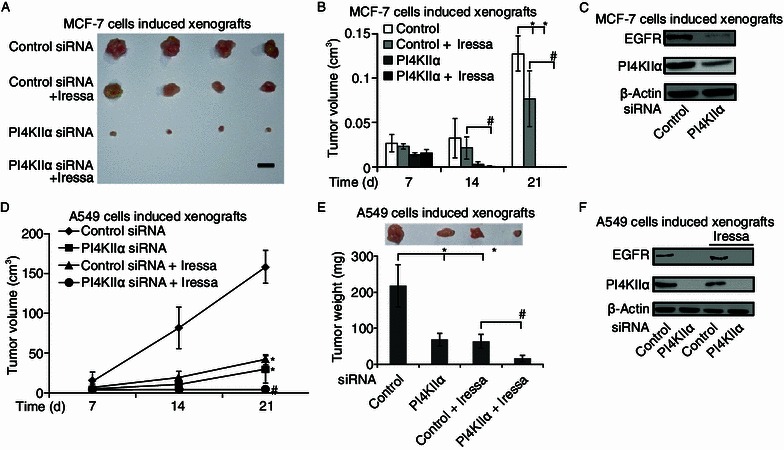
Enhancing effects of PI4KIIα knockdown on anti-tumor activity of the EGFR inhibitor Iressa *in vivo*. BALB/c nude mice were injected subcutaneously. The mice were treated with Iressa (50 mg/kg, 5/7 days, ig.) or vehicle for 2 weeks from the 7th day after tumor transplantation. Tumor growth was monitored and was shown as mean volumes ± SD, *P < 0.05, as compared to control RNAi tumors, ^#^*P* < 0.05 as compared to combined tumor treatment with both Iressa and control RNAi. Effect of single or dual kinase repression on MCF-7 tumor growth (A and B) and A549 tumor growth (D and E). Protein levels of PI4KIIα and EGFR in MCF-7 cell-induced tumor (C) and A549 cell-induced tumors (F) were analyzed by Western blotting. All results presented above represent data from two independent experiments

To determine whether simultaneous inhibition of PI4KIIα and EGFR exhibited similar enhanced effects *in vivo*, xenograft models were used. As shown in Fig. 4A and 4B, both Iressa (an EGFR targeted anti-tumor drug) treatment and PI4KIIα knockdown inhibited MCF-7 tumor growth, which is consistent with our previous results showing that downregulation of PI4KIIα results in nearly complete inhibition of MCF-7 cell-induced tumor growth *in vivo* (Li et al., 2010). Following regular drug administration, Iressa alone showed about 47% inhibition of tumor growth as assessed by tumor volume. In comparison, when Iressa treatment was combined with PI4KIIα knockdown, tumor almost completely disappeared during the same time period. EGFR is known to be an important anti-tumor target for NSCLC treatment (Harandi et al., 2009). Therefore, we investigated whether the cooperative therapeutic effects of PI4KIIα and EGFR inhibition also exist for A549-induced tumor. As shown in Fig. 4D and 4E, treatment with Iressa inhibited A549 cell-induced tumor growth by about 74%. In contrast, upon PI4KIIα knockdown in combination with Iressa, the A549 cell-induced tumor exhibited negligible signs of growth. Combined inhibition of PI4KIIα and EGFR displayed far superior anti-tumor traits than single drug use, both in terms of tumor volume and weight. Next, we detected PI4KIIα and EGFR expression in these tumors, and the results indicated that the expression level of EGFR was markedly decreased in PI4KIIα RNAi cell-induced tumors in both types of xenograft models (Fig. 4C and 4F).

### The mechanisms of EGFR regulation by PI4KIIα

**Figure 5:**
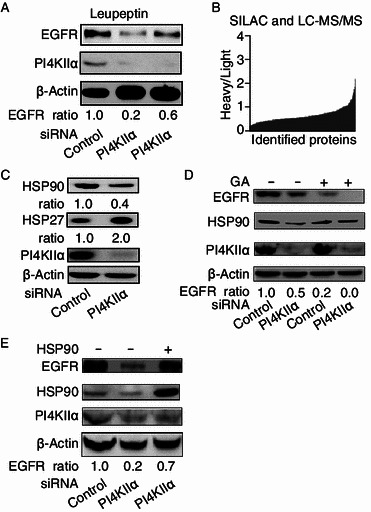
Mechanisms for regulation of EGFR expression upon PI4KIIα knockdown. MCF-7 cells were transfected with control or PI4KIIα siRNA unless indicated otherwise. (A) Effect of inhibition of lysosomal degradation on the regulation of PI4KIIα on EGFR expression. Cells were treated with 10 μmol/L Leupeptin for 6 h, except controls. (B) Proteins affected by PI4KIIα knockdown in MCF-7 cells identified with SILAC and LC-MS/MS. (C) Regulation of PI4KIIα on HSP90 and HSP27. (D) Enhanced effects following combinatorial treatment with both HSP90 inhibitor geldanamycin (GA) and PI4KIIα siRNA on EGFR protein levels. PI4KIIα siRNA-treated cells and control cells were incubated with 50 μmol/L geldanamycin (GA) for 24 h, except control experiments, and proteins measured by Western blot. (E) Effect of overexpression of HSP90 on EGFR expression following PI4KIIα knockdown. HSP90 or control vectors were transfected into PI4KIIα siRNA-treated or control cells for 36 h, and indicated proteins detected by Western blot, with β-actin serving as control. All results presented, except those for LC-MS/MS experiments, represent data from three independent experiments

To understand the precise molecular mechanisms of how PI4KIIα regulates EGFR levels, we first analyzed EGFR transcription levels by RT-PCR, and no difference was found between the control cells and PI4KIIα RNAi cells (Fig. S2A and S2B). We then tested the effect of PI4KIIα knockdown on EGFR degradation. Leupeptin (lysosome inhibitor, Fig. 5A) was added into the PI4KIIα RNAi-treated cells, and it can partly rescue the effect of PI4KIIα knockdown on EGFR protein level. These results suggest that PI4KIIα regulates EGFR at least partially through the lysosome degradation pathway. To determine the molecules mediating the downregulation of EGFR, we performed quantitative proteomic analysis by employing stable isotope labeling with amino acids in cell culture (SILAC) in combination with LC-MS/MS. Samples were prepared as described in the MATERIALS AND METHODS section and as shown in Fig. S3. A total of 252 targets were identified on the basis of the LC-MS/MS results (Fig. 5B; Table S1). For instance, the heavy/light ratio of the heat shock protein HSP90AB1 (NM_007355) was approximately 0.52, indicating that PI4KIIα knockdown results in downregulation of HSP90AB1 protein levels. An earlier report showed that HSP90 interacts with EGFR and prevents its degradation (Sawai et al., 2008; Ahsan et al., 2012), we therefore wondered whether HSP90 is providing a functional link between PI4KIIα and EGFR. To this end, we firstly confirmed the MS result by Western blot. As shown in Fig. 5C, HSP90 was significantly downregulated in PI4KIIα RNAi-treated cells. Furthermore, HSP27 was observed to be upregulated, which is consistent with its assumed role as a downstream target of HSP90 (McCollum et al., 2006). Furthermore, enhanced effects of geldanamycin (GA, a specific inhibitor of HSP90) and PI4KIIα siRNA on the induction of ligand-free EGFR degradation were detected (Fig. 5D). Overexpression of HSP90AB1 in PI4KIIα RNAi-treated MCF-7 cells rescued the effects of PI4KIIα knockdown on EGFR (Fig. 5E). Thus, it can be assumed that the physiological role of PI4KIIα is at least partly dependent on HSP90.

### Oncogenes regulated by combined PI4KIIα and EGFR inhibition

**Figure 6:**
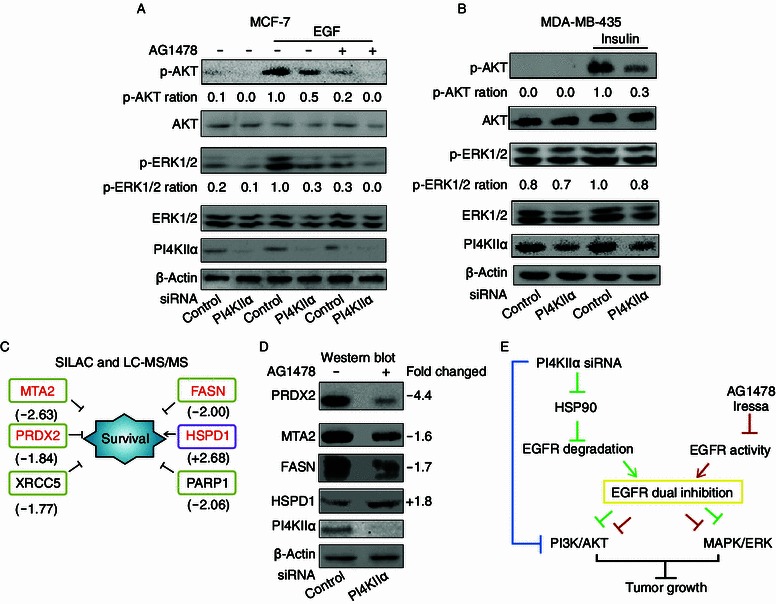
Signaling pathways and oncogenes affected by the combinatorial inhibition of both PI4KIIα and EGFR. Cells were transfected with control or PI4KIIα siRNA for 72 h, (A) effect of PI4KIIα knockdown and AG1478 on p-AKT and p-ERK1/2 in MCF-7 cells. MCF-7 cells were incubated with 100 nmol/L AG1478 for 2 h followed by addition of EGF for 10 min as indicated, except for controls. (B) Effect of PI4KIIα knockdown and AG1478 treatment on p-AKT and p-ERK1/2 in MDA-MB-435 cells. MDA-MB-435 cells were incubated with 100 μg insulin for 10 min, except controls. (C) Molecules affected by combinatorial inhibition of PI4KIIα and EGFR in MCF-7. Numbers below protein names indicate fold changes as identified by SILAC and LC-MS/MS method. (D) Western blot validation of proteins identified by SILAC and LC-MS/MS. (E) Model of the effects on tumor growth upon combinatorial inhibition of EGFR and PI4KIIα

It is well known that EGFR and PI4KIIα both can regulate PI3K and MAPK signaling cascades (Chu et al., 2010; Li et al., 2010; Zhang et al., 2012), therefore we wondered if the enhanced inhibitory effects observed may result from the involvement of these two pathways. To this end, we tested the effects of PI4KIIα knockdown on both PI3K/AKT and MAPK/ERK pathways in two different cell lines, the EGFR-positive MCF-7 cells and the EGFR-negative MDA-MB-453 cells (Fig. S2B). In the MCF-7 cells, PI4KIIα knockdown suppressed both p-AKT and p-ERK1/2 level. When combined with an AG1478 treatment, the observed inhibitory effects were markedly enhanced (Fig. 6A). In contrast, in MDA-MB-453 cells, PI4KIIα knockdown only decreased the level of p-AKT, but not those of p-ERK (Fig. 6B), indicating that the effect of PI4KIIα on the MAPK pathway depends on EGFR function. It is well established that both PI3K and MAPK signaling pathways play important roles in regulation of a plethora of genes associated with tumor progression. In order to understand the precise gene targets involved in our combinatorial inhibition approach, we performed a quantitative comparison of protein targets in MCF-7 cells treated with or without combination treatment (PI4KIIα siRNA + 10 μmol/L AG1478) by employing SILAC and LC-MS/MS (Fig. S3). A total of 348 proteins were identified (Table S2). We found, for instance, that 6 cancer-related targets changed their expression levels: HSPD1 (Ghosh et al., 2008), PARP1 (Cipak and Jantova, 2010), XRCC5 (Yang et al., 2008), PRDX2 (Stresing et al., 2013), MTA2 (Cui et al., 2006) and FASN (Zhou et al., 2003) (Fig. 6C). Four of them were validated by Western blot (Fig. 6D). With the exception of HSPD1, all targets were downregulated upon combinatorial treatment, suggesting that it can suppress cancer cell survival by affecting the expression levels of these validated cancer-related target genes (Fig. 6C). In conclusion, our results indicate that PI4KIIα knockdown reduces EGFR protein level in a degradation-dependent pathway, and the dual inhibition of EGFR protein level by PI4KIIα RNAi and EGFR activity by its specific small molecular inhibitors exerts superior outcomes for tumor therapy.

## Discussion

Cancer progression is an orchestrated process that is regulated by multiple oncogenes and tumor suppressors, rendering single-target therapy prone to be of low effectiveness and raise the resistance of tumors to cancer treatment. To overcome these drawbacks, combinatorial treatment involving multiple targets is an absolute imperative (Koppikar et al., 2008; Ren et al., 2008; Martelli et al., 2012). In this study, we found that PI4KIIα could be an ideal combinatorial target for EGFR treatment via regulating EGFR degradation. Based on our experimental results, we propose a model for the combinatorial inhibition of EGFR and PI4KIIα: First, PI4KIIα knockdown inhibits the PI3K/AKT pathway directly (Fig. 6E, blue lines). Second, PI4KIIα promotes the degradation of EGFR in HSP90-dependent pathway, and the reduced EGFR expression then affects the activity of HER-2, and further suppresses both PI3K/AKT and MAPK signaling pathways (green lines). Third, EGFR inhibitors can further enhance weakening of the PI3K/AKT and MAPK signaling pathways (red lines). Therefore, combined inhibition of PI4KIIα and EGFR showed tripled anti-tumor effect (Fig. 6E). To our knowledge, this is the first report to shed a light on the superiority of the combined inhibition of PI4KIIα and EGFR as a new therapeutic anti-tumor strategy.

To date, EGFR is one of the most successful rational drug targets in clinical cancer therapy (Flynn et al., 2009). The clinical efficacy of anti-EGFR therapy has been confirmed for a growing number of cancer types, including breast, colon, pancreas, head and neck, renal and lung carcinomas (Harandi et al., 2009). Therefore, the discovery of the specific regulator of EGFR should yield important insights for improved clinical application. At present, two main anti-EGFR approaches have been pursuit for clinical development, namely the use of monoclonal antibodies (mAb, e.g. Cetuximab and Panitumumab) and small molecular tyrosine kinase inhibitors (smTKIs, e.g.: Gefitinib, Erlotinib and Lapatinib). Antibodies compete with growth factor ligands and result in EGFR internalization and degradation, whilst the kinase inhibitors compete with ATP for binding to the tryrosine kinase portion of the receptor (Imai and Takaoka, 2006; Harandi et al., 2009). However, both approaches exhibit serious limitations: the effect of smTKIs depends on the presence of certain mutant form of EGFR (For example: the single-point substitution mutation L858R in exon 21), however, this mutant form is only present in approximately 30%–50% of Asians and 10% of non-Asians (Shim et al., 2011; Kerr, 2013). Moreover, the efficacy of smTKIs in combination with chemotherapy or radiotherapy has been shown to be very low (Giaccone et al., 2004; Herbst et al., 2004; Harandi et al., 2009). The anti-tumor effects of mAbs are restricted to only a fraction of patients, and also will disappear if the extracellular domain of EGFR becomes mutated during the genetic development of the tumor. Besides, mAbs drugs show several additional weaknesses in their clinical application: expensive, only be administered intravenously, and low efficiency in terms of tissue penetration, tumor retention and blood clearance (Imai and Takaoka, 2006). Thus, there is urgent clinical need to develop novel anti-EGFR approaches with improved therapeutic characteristics.

The effects of dual-reagent targeting of EGFR have been examined previously using pre-clinical models. Huang et al. (Huang et al., 2004) and Matar et al. (Matar et al., 2004) reported that Cetuximab (a mAb that causes EGFR degradation) in combination with either Gefitinib or Erlotinib showed enhanced inhibition of tumor cell growth when compared to the effects of either agent alone. Two other agents, for example, an HSP90 inhibitor and Gemcitabine, also enhance the effects of EGFR targeted smTKIs (Feng et al., 2007; Bartholomeusz et al., 2011; Ahsan et al., 2012; Xu et al., 2012). It is worth noting that all of the above-mentioned reagents share one feature with siRNA-induced PI4KIIα knockdown, namely promoting EGFR degradation (Feng et al., 2007; Harandi et al., 2009; Ahsan et al., 2012). We therefore predict that the enhanced effects on EGFR inhibition may be obtained not only through PI4KIIα or HSP90, but also through activation of other elements that induce EGFR degradation, for example Caspase 3 and Pnck activators (He et al., 2006; Deb et al., 2011). The dual suppression of EGFR protein and activity presents a promising avenue for EGFR-targeted therapies, with the advantage of improving efficiency, reducing side effects and lowering the effective dosage, while at the same time minimizing resistance. Taking these points into account, PI4KIIα is undoubtedly an ideal target for tumor therapy: (i) PI4KIIα knockdown increases EGFR degradation in a subtype-specific manner, since PI4KIIα is just one subtype within the PI4K family, specific downregulation of PI4KIIα is unlikely to induce complicated side effects, as other PI4K family members remain functional. Recently, Simons et al. have shown that mice with heterozygous deficiency of PI4KIIα do not display effects on life span (Simons et al., 2009). (ii) Apart from its effect on EGFR degradation, PI4KIIα inhibition directly interferes with the PI3K/AKT pathway, thus the combined inhibition of PI4KIIα and EGFR results in a triple anti-tumor effect (Fig. 6E).

In conclusion, we investigated the pivotal role of PI4KIIα in the regulation of EGFR protein levels, and our functional studies indicate that PI4KIIα represents a promising therapeutic target that could be used in combination with existing EGFR treatments used in breast cancer and NSCLC therapy. We propose that the inhibition of EGFR at the levels of expression and activity should be used as a new strategy, as it exhibits far superior results for EGFR-dependent tumor inhibition. Up to now, we have resolved the crystal structure of PI4KIIα (Zhou et al., 2014) and subtype-specific PI4KIIα inhibitor candidates based on rational design are currently screened for mimicking the effects observed here for PI4KIIα siRNA knockdown. Furthermore whether the inhibition of PI4KIIα and HER-2 also exits the combinatorial anti-tumor effect will be investigated in the future.

## Materials and methods

### Reagent, plasmids and antibodies

AG1478, and geldanamycin were purchased from R&D Systems (Minneapolis, USA). Phosphatidylinositol 4-phosphate (PI4P) and Carrier 3 were purchased from Echelon Biosciences (Utah, USA). Human HSP90AB1 plasmid was kindly provided by Professor Fei Sun (Institute of Biophysics, Chinese Academy of Sciences, China) and was ligated into pcDNA3.1 (Invitrogen, Paisley, UK) vector for expression. Antibodies to PRDX2, MTA2, FASN and HSPD1 were purchased from Abcam (Cambridge, UK). Rabbit polyclonal PI4KIIα antibody was a kind gift from Pietro De Camilli (Yale University, HHMI) (Guo et al., 2003). Antibodies to HER-2, p-HER-2 (Tyr1248), EGFR and β-actin were purchased from Santa Cruz (Texas, USA). AKT antibody, p-AKT antibody, ERK antibody and p-ERK antibody were from Cell Signaling Technology (Herts, UK). SILAC DMEM was purchased from Thermo Fisher Scientific (New Hampshire, USA). [^12^C_6_,^14^N_2_]-Lys, [^12^C_6_,^14^N_4_]-Arg, [^13^C_6_,^15^N_2_]-Lys and [^13^C_6_,^15^N_4_]-Arg were purchased from Cambridge Isotope Laboratories (Massachusetts, USA). Other reagents were purchased from Sigma (Dorset, UK) unless otherwise stated.

### Cell culture, transfection and siRNA interference

All cells except MDA-MB-231 were cultured in a humidified atmosphere in the presence of 5% CO_2_ and 95% air at 37°C, and the MDA-MB231 cells cultured in L15 medium in 100% air at 37°C. For EGF stimulation, the cells were incubated in 100 ng/mL EGF for 10 min. Cells were transiently transfected with plasmids using Lipofectamine 2000 reagent (Invitrogen) according to the manufacturer’s instructions. For siRNA, the sequences targeting human PI4KIIα (GenBank accession number NM_018425) PI4KIIα1 spans nucleotides 888–908, and PI4KIIα2 spans 494–512, both are specific for hPI4KIIα (Wang et al., 2003; Pan et al., 2008). hPI4KIIβ (GenBank accession number NM_018323.3) siRNA spans nucleotides 384–403 is specific for hPI4KIIβ. A non-targeting siRNA (Thermotogameritimia siRNA, UUCUCCGAACGUGUCACGUTT) was used as a negative control.

### Human cancer specimens and original isolated breast cancer cells

Patient breast cancer tissues were obtained from Xuanwu Hospital (Capital Medical University, Beijing, China). The 43 breast cancer tissues were lysed by RIPA, and the protein concentration was determined using a BCA™ protein assay kit. The protein solution was adjusted to 1.5 mg/mL, and 30 μg protein was used for SDS-PAGE/Western blot. Using the levels observed for patient No. 27 as standards for PI4KIIα and EGFR expression, all the PI4KIIα and EGFR immunoblotting results were quantified, and the changes expressed as log_2_ values. Two tumor specimen, used for isolating primary isolated breast cancer cells (PIBC) arrived at the laboratory within 30 min of surgery and immediately mechanically disaggregated. And single cell suspension was prepared as described previously with small modifications (Ponti et al., 2005). Briefly, tissue fragments were incubated at 37°C for 2 h in a 1:1 solution of collagenase/hyaluronidase. After filtration through a 30 μm pore filter, cells were seeded at 1,000 cells/mL in DMEM supplemented with 10% fetal bovine serum. After culturing for 6 generations, cells were ready for use for experiments. All patients gave consent to the use of their tissues for research projects.

### Animal studies

Six-to-eight-week old male BALB/c nude mice (purchased from Weitonglihua, Beijing, China) were allowed to acclimatize for 1 week under specific pathogen-free conditions in the animal facility of the Institute of Biophysics, Chinese Academy of Sciences. All procedures involving animals and their care were approved by the animal ethics committee of Institute of biophysics, Chinese Academy of Sciences. For each mouse 200 μL of a PI4KIIα RNAi-treated cell suspension or control cells (3 × 10^7^ cells/mL MCF-7 cells or 1 × 10^7^ cells/mL A549 cells) were subcutaneously injected into BALB/c nude mice (4 mice per group), with control cells in the left side and PI4KIIα RNAi cells in the right side of the venter region. After 7 days of growth, the mice were divided into two groups, one group was treated with Iressa (50 mg/kg, 5/7 days, ig.) and the other group was treated with PBS for 2 weeks, and the tumor growth was monitored every 7 days. The tumor volumes were calculated for live mice, and the mice were then sacrificed (Li et al., 2010), the tumor tissues were isolated on the day indicated for tumor weight measurement, and lastly the PI4KIIα and EGFR protein levels were detected by Western blotting.

### SILAC labeling, protein separation and identification

SILAC labeling was carried out according to a previously published method with minor modifications (Zhou et al., 2010). Briefly, the light medium was made by mixing SILAC DMEM with 100 mg/mL [^12^C_6_,^14^N_2_]-Lys, 100 mg/mL [^12^C_6_,^14^N_4_]-Arg, 10% dialyzed fetal bovine serum and 100 U/mL penicillin and 100 µg/mL streptomycin. The heavy medium had the same composition except that Lys and Arg were substituted by [^13^C_6_,^15^N_2_]-Lys and [^13^C_6_,^15^N_4_]-Arg. MCF-7 cells were cultured in the light or heavy isotope labeled medium in a humidified atmosphere with 5% CO_2_ in air at 37°C for 13 generations in order to get effective incorporation (>95%). Light isotope labeled MCF-7 cells were transfected with control siRNA (Sample 1 and Sample 3), while the heavy isotope labeled MCF-7 cells were divided into two groups, one was transfected with PI4KIIα siRNA for 96 h (Sample 2), and the other group was transfected with PI4KIIα siRNA for 96 h combined with 10 μmol/L AG1478 treatment for 72 h (Sample 4). 10^6^ MCF-7 cells labeled with heavy isotope were then counted by flow cytometry, and mixed with equal amounts of cells labeled with light isotope. Nuclear-Cytosol Extraction kit (PPLYGEN, China) was used for the isolation of cell components, Membrane (M), Nucleus (N) and Cytoplasm (C), after SDS-PAGE of the extracted proteins, all bands were cut out and digested in-gel according to a previously published method (Shevchenko et al., 2006). Peptides were extracted into acetonitrile and were used for analysis with LC-MS/MS (Zhang et al., 2010).

### PI4P delivery

PI4P was pre-mixed with Carrier 3 (Echelon Biosciences Inc.) at 1 to 1 molar ratio for a final concentration of (500 μmol/L) at RT for 10 min and added to MCF-7 cells at a final concentration of 50 μmol/L. After 6 h incubation at room temperature, 100 ng/mL EGF was added or not as indicated for 10 min, and the autophosphorylation level of HER-2 at Tyr1248 or EGFR protein level was evaluated by Western blot.

### Statistical analysis

Statistical analysis was performed using the two-tailed paired Student’s *t* test. We considered data statistically significant when the *P* value was <0.05 or <0.01 as indicated in the legends. All data are expressed as mean ± SD. The correlation between PI4KIIα and EGFR expression was analyzed by Pearson correlation coefficient, we considered the strength of association between the variables is high when *r* > 0.6 and data statistically significant when *P* < 0.05.

## Electronic supplementary material

Below is the link to the electronic supplementary material.

Supplementary material 1 (PDF 426 kb)

## Abbreviations

GA: geldanamycin
INPP4B: inositol polyphosphate 4-phosphatase type II
mAb: monoclonal antibodies
NSCLC: non-small-cell lung carcinoma
PI4KIIα: phosphatidylinositol 4-kinase IIα
PI: phosphatidylinositol
PI4P: phosphatidylinositol 4-phosphate
PIBC: primary isolated breast cancer cells
smTKIs: small molecular tyrosine kinase inhibitors
SILAC: stable isotope labeling with amino acids in cell culture
