# Supplementary material for: Dual inhibition of EGFR at protein and activity level via combinatorial blocking of PI4KIIα as anti-tumor strategy
Source: Protein Cell. 2014 May 7;5(6):457–68. doi: 10.1007/s13238-014-0055-y (PMC4026421; doi:10.1007/s13238-014-0055-y)
Supplement: Supplementary file 1 — Supplementary material 1 (PDF 426 kb) [file 13238_2014_55_MOESM1_ESM.pdf]

Supporting Information

Dual inhibition of EGFR at protein and activity level via combinatorial blocking of PI4KIIα as anti-tumor strategy

Jiangmei Li[1], Lunfeng Zhang[1,2], Zhen Gao[1,2], Hua Kang[3], Guohua Rong[3],Xu Zhang[4], Chang Chen[1]*

[1] National Laboratory of Biomacromolecules, Institute of Biophysics, Chinese Academy of Sciences, Beijing, China;

[2] University of Chinese Academy of Sciences, Beijing, China

[3] Department of General Surgery, Xuanwu Hospital, Capital Medical University, Beijing, China;

[4] Tianjin Research Center of Basic Medical Science, Tianjin Medical University, Tianjin, China;

*Correspondence should be addressed to: Chang Chen, National Laboratory of Biomacromolecules, Institute of Biophysics, Chinese Academy of Sciences, 15 Datun Road, Chaoyang District, Beijing 100101, PR China. E-mail: changchen@moon.ibp.ac.cn. Tel：86-010-64888406. Fax: 86-010-64871293

Running Title: PI4KIIα, an ideal combinatorial target for EGFR treatment

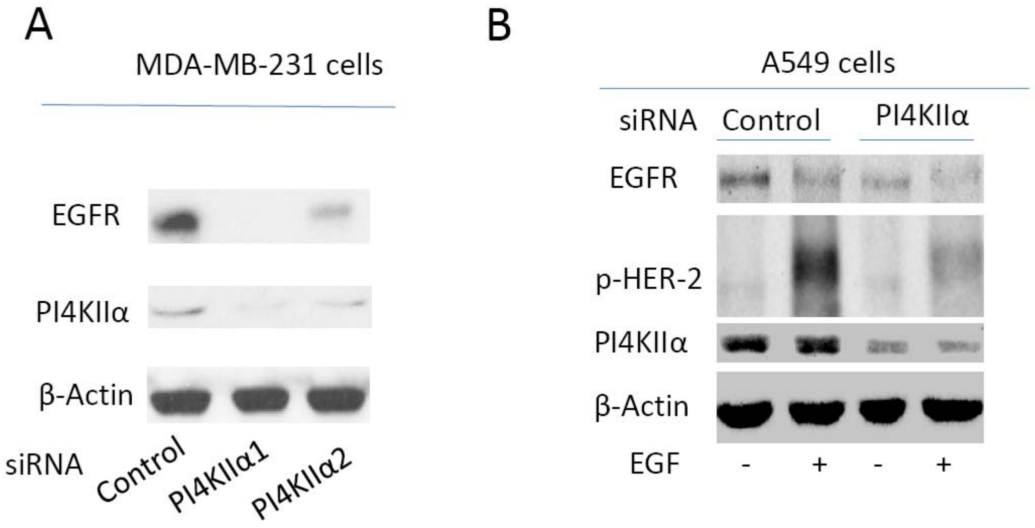

Figure S1. **Effect of PI4KIIα knockdown in different cell lines on EGFR protein levels. A)** MDA-MB-231 cells. **B)** A549 cells. Cells were transfected with either control siRNA or PI4KIIα siRNA (PI4KIIα 1 and PI4KIIα 2 refer to siRNA targeting different site of the PI4KIIα mRNA) for 72 hours, and then treated 100 ng/ml EGF for 10 minutes, except controls, and proteins indicated measured by Western blot. All results presented above represent data from three independent experiments.

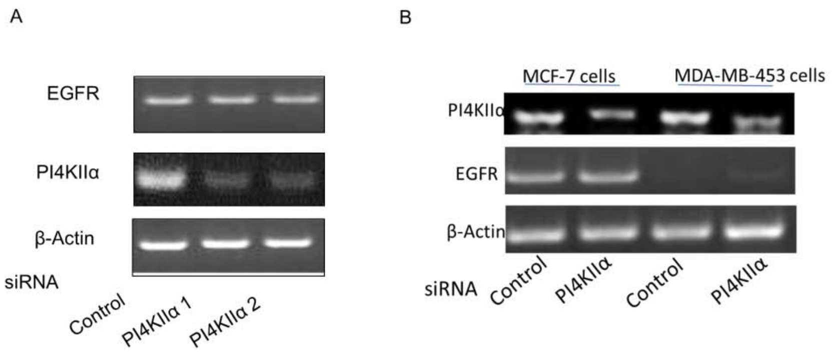

Figure S2. PI4KIIα knockdown not affect EGFR transcription level. A) MCF-7 cells were transfected with control siRNA or two different PI4KIIα siRNAs for 72 hours. mRNA levels of EGFR and PI4KIIα. B) MCF-7 cells and MDA-MB-435 cells were transfected with control or PI4KIIα siRNA for 72 hours, and levels of EGFR and PI4KIIα mRNA measured.

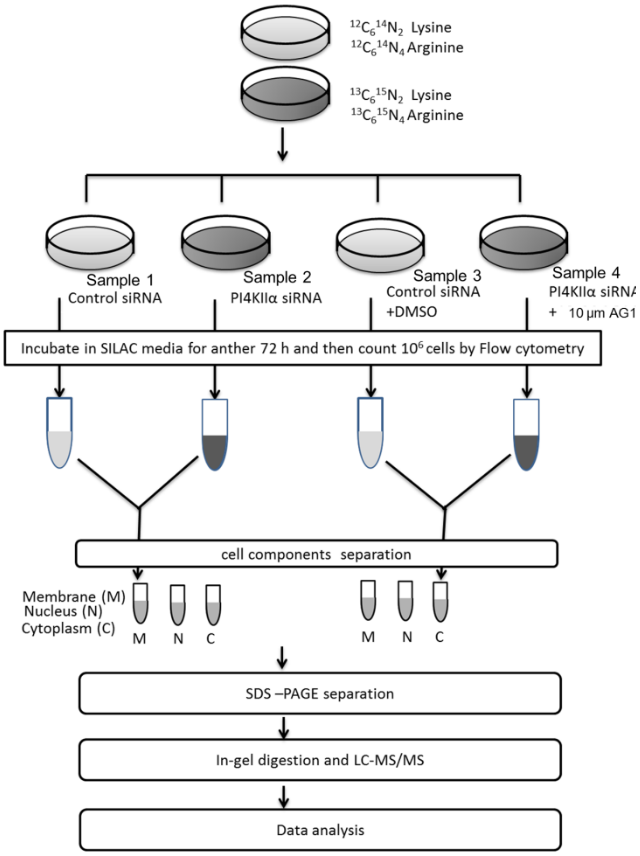

Figure S3. **Proteomics analysis of molecular networks affected by PI4KIIα knockdown or dual inhibition by SILAC and LC-MS/MS.** Schematic flowchart for the discovery of molecular networks regulated by PI4K and EGFR dual inhibition in MCF-7 cells. Detailed information was described in method section.

Table S1. Protein targets identified by SILAC and LC-MS/MS for control RNAi MCF-7 cells (sample 1) and PI4KIIα RNAi MCF-7 cells (sample 2).

Accession	Description	Heavy/Light*
**O94776**	Metastasis-associated protein MTA2	0.22
**P04040**	Catalase	0.24
**P14923**	Junction plakoglobin	0.26
**Q08945**	FACT complex subunit SSRP1	0.26
**Q9UMS4**	Pre-mRNA-processing factor 19	0.26
**P12270**	Nucleoprotein TPR	0.26
**P08670**	Vimentin	0.27
**P09874**	Poly [ADP-ribose] polymerase 1	0.28
**Q9BTV4**	Transmembrane protein 43	0.29
**Q14690**	Protein RRP5 homolog	0.30
**Q15269**	Periodic tryptophan protein 2 homolog	0.31
**P14866**	Heterogeneous nuclear ribonucleoprotein L	0.32
**P23246**	Splicing factor, proline- and glutamine-rich	0.32
**Q9BQ39**	ATP-dependent RNA helicase DDX50	0.33
**O43143**	Putative pre-mRNA-splicing factor ATP-dependent RNA helicase DHX15	0.34
**Q13151**	Heterogeneous nuclear ribonucleoprotein A0	0.34
**O75533**	Splicing factor 3B subunit 1	0.35
**H0Y8X1**	Succinate dehydrogenase [ubiquinone] flavoprotein subunit, mitochondrial	0.35
**H0YDK2**	Histone-binding protein RBBP4 (Fragment)	0.35
**Q99436**	Proteasome subunit beta type-7	0.35
**P36871**	Phosphoglucomutase-1	0.36
**Q9NVP1**	ATP-dependent RNA helicase DDX18	0.36
**P35237**	Serpin B6	0.37
**Q13347**	Eukaryotic translation initiation factor 3 subunit I	0.38
**P30740**	Leukocyte elastase inhibitor	0.38
**P48147**	Prolyl endopeptidase	0.39
**E9PBF6**	Lamin-B1	0.39

**Q08211**	ATP-dependent RNA helicase A	0.39
**P12004**	Proliferating cell nuclear antigen	0.40
**P04080**	Cystatin-B	0.40
**Q96GQ7**	Probable ATP-dependent RNA helicase DDX27	0.40
**P14550**	Alcohol dehydrogenase [NADP(+)]	0.40
**Q16795**	NADH dehydrogenase [ubiquinone] 1 alpha subcomplex subunit 9, mitochondrial	0.41
**P24539**	ATP synthase subunit b, mitochondrial	0.42
**O15427**	Monocarboxylate transporter 4	0.42
**Q8TDN6**	Ribosome biogenesis protein BRX1 homolog	0.42
**P32119**	Peroxiredoxin-2	0.43
**P22087**	rRNA 2'-O-methyltransferase fibrillarin	0.43
**P05386**	60S acidic ribosomal protein P1	0.43
**Q15645**	Pachytene checkpoint protein 2 homolog	0.43
**O00487**	26S proteasome non-ATPase regulatory subunit 14	0.44
**Q6S8J3**	POTE ankyrin domain family member E	0.44
**Q09666**	Neuroblast differentiation-associated protein AHNAK	0.45
**P16152**	Carbonyl reductase [NADPH] 1	0.45
**P28072**	Proteasome subunit beta type-6	0.45
**Q7KZF4**	Staphylococcal nuclease domain-containing protein 1	0.45
**P52907**	F-actin-capping protein subunit alpha-1	0.45
**P52292**	Importin subunit alpha-2	0.45
**P25787**	Proteasome subunit alpha type-2	0.46
**P08574**	Cytochrome c1, heme protein, mitochondrial	0.46
**P24534**	Elongation factor 1-beta	0.46
**Q15393**	Splicing factor 3B subunit 3	0.46
**P28066**	Proteasome subunit alpha type-5	0.47
**P26447**	Protein S100-A4	0.47
**P37802**	Transgelin-2	0.47
**O75874**	Isocitrate dehydrogenase [NADP] cytoplasmic	0.47
**P20618**	Proteasome subunit beta type-1	0.48
**Q9Y2X3**	Nucleolar protein 58	0.48

**P62269**	40S ribosomal protein S18	0.48
**O95678**	Keratin, type II cytoskeletal 75	0.48
**P80723**	Brain acid soluble protein 1	0.48
**Q14566**	DNA replication licensing factor MCM6	0.48
**P00966**	Argininosuccinate synthase	0.48
**P22314**	Ubiquitin-like modifier-activating enzyme 1	0.48
**P28070**	Proteasome subunit beta type-4	0.48
**O75643**	U5 small nuclear ribonucleoprotein 200 kDa helicase	0.49
**P13010**	X-ray repair cross-complementing protein 5	0.49
**Q6P2Q9**	Pre-mRNA-processing-splicing factor 8	0.49
**P49915**	GMP synthase [glutamine-hydrolyzing]	0.49
**P30041**	Peroxiredoxin-6	0.49
**E9PBV3**	Suprabasin	0.50
**P53985**	Monocarboxylate transporter 1	0.50
**Q99497**	Protein DJ-1	0.50
**P34932**	Heat shock 70 kDa protein 4	0.50
**P41091**	Eukaryotic translation initiation factor 2 subunit 3	0.50
**Q96CS3**	FAS-associated factor 2	0.50
**P78527**	DNA-dependent protein kinase catalytic subunit	0.51
**Q9NQR4**	Omega-amidase NIT2	0.51
**Q9BYZ2**	L-lactate dehydrogenase A-like 6B	0.51
**O00116**	Alkyldihydroxyacetonephosphate synthase, peroxisomal	0.51
**O43242**	26S proteasome non-ATPase regulatory subunit 3	0.51
**P08238**	Heat shock protein HSP 90-beta	0.51
**P49257**	Protein ERGIC-53	0.52
**O95373**	Importin-7	0.52
**P31930**	Cytochrome b-c1 complex subunit 1, mitochondrial	0.52
**P30084**	Enoyl-CoA hydratase, mitochondrial	0.53
**P61353**	60S ribosomal protein L27	0.53
**P17931**	Galectin-3	0.53
**P25705**	ATP synthase subunit alpha, mitochondrial	0.53

**P52597**	Heterogeneous nuclear ribonucleoprotein F	0.53
**Q9BWD1**	Acetyl-CoA acetyltransferase, cytosolic	0.53
**P62081**	40S ribosomal protein S7	0.53
**P25398**	40S ribosomal protein S12	0.53
**P60903**	Protein S100-A10	0.54
**P62854**	40S ribosomal protein S26	0.54
**P48637**	Glutathione synthetase	0.54
**P46777**	60S ribosomal protein L5	0.54
**E9PK82**	40S ribosomal protein S3 (Fragment)	0.54
**O14980**	Exportin-1	0.54
**P43121**	Cell surface glycoprotein MUC18	0.54
**O43707**	Alpha-actinin-4	0.54
**Q14126**	Desmoglein-2	0.54
**Q9BVC6**	Transmembrane protein 109	0.55
**O75131**	Copine-3	0.55
**P08754**	Guanine nucleotide-binding protein G(k) subunit alpha	0.55
**P23526**	Adenosylhomocysteinase	0.55
**P62906**	60S ribosomal protein L10a	0.55
**P10599**	Thioredoxin	0.55
**P49721**	Proteasome subunit beta type-2	0.56
**P37837**	Transaldolase	0.56
**Q6ZS74**	Ras-related protein Ral-B	0.56
**P00491**	Purine nucleoside phosphorylase	0.56
**P40429**	60S ribosomal protein L13a	0.56
**P30050**	60S ribosomal protein L12	0.57
**Q08380**	Galectin-3-binding protein	0.57
**P26373**	60S ribosomal protein L13	0.57
**P27348**	14-3-3 protein theta	0.57
**P17301**	Integrin alpha-2	0.58
**P62701**	40S ribosomal protein S4, X isoform	0.58
**P31947**	14-3-3 protein sigma	0.58

**P15121**	Aldose reductase	0.59
**P08708**	40S ribosomal protein S17	0.59
**P62266**	40S ribosomal protein S23	0.59
**P32970**	CD70 antigen	0.59
**Q01650**	Large neutral amino acids transporter small subunit 1	0.59
**P21266**	Glutathione S-transferase Mu 3	0.59
**Q14151**	Scaffold attachment factor B2	0.59
**P50914**	60S ribosomal protein L14	0.59
**Q16719**	Kynureninase	0.60
**Q9BVK6**	Transmembrane emp24 domain-containing protein 9	0.60
**P46782**	40S ribosomal protein S5	0.60
**Q9BS26**	Endoplasmic reticulum resident protein 44	0.60
**P38919**	Eukaryotic initiation factor 4A-III	0.60
**Q99714**	3-hydroxyacyl-CoA dehydrogenase type-2	0.60
**P35268**	60S ribosomal protein L22	0.60
**P46781**	40S ribosomal protein S9	0.60
**P31153**	S-adenosylmethionine synthase isoform type-2	0.61
**P51571**	Translocon-associated protein subunit delta	0.61
**P46778**	60S ribosomal protein L21	0.62
**P00403**	Cytochrome c oxidase subunit 2	0.62
**P62280**	40S ribosomal protein S11	0.63
**P52888**	Thimet oligopeptidase	0.63
**P06703**	Protein S100-A6	0.63
**P19338**	Nucleolin	0.63
**O00299**	Chloride intracellular channel protein 1	0.63
**P62249**	40S ribosomal protein S16	0.63
**P84243**	Histone H3.3	0.63
**Q9Y3U8**	60S ribosomal protein L36	0.63
**Q8NFJ5**	Retinoic acid-induced protein 3	0.64
**P00492**	Hypoxanthine-guanine phosphoribosyltransferase	0.64
**P61586**	Transforming protein RhoA	0.64

**Q12905**	Interleukin enhancer-binding factor 2	0.64
**P26038**	Moesin	0.65
**Q00839**	Heterogeneous nuclear ribonucleoprotein U	0.65
**P07814**	Bifunctional glutamate/proline--tRNA ligase	0.66
**Q99650**	Oncostatin-M-specific receptor subunit beta	0.66
**P05198**	Eukaryotic translation initiation factor 2 subunit 1	0.66
**H7BZJ3**	Thioredoxin (Fragment)	0.66
**P49755**	Transmembrane emp24 domain-containing protein 10	0.66
**Q562R1**	Beta-actin-like protein 2	0.66
**P62263**	40S ribosomal protein S14	0.67
**Q07021**	Complement component 1 Q subcomponent-binding protein, mitochondrial	0.67
**I3L4N8**	Actin, cytoplasmic 2 (Fragment)	0.68
**P58107**	Epiplakin	0.68
**P13667**	Protein disulfide-isomerase A4	0.68
**P14625**	Endoplasmin	0.68
**P13639**	Elongation factor 2	0.69
**Q96AG4**	Leucine-rich repeat-containing protein 59	0.69
**P35579**	Myosin-9	0.71
**F5H867**	4F2 cell-surface antigen heavy chain (Fragment)	0.71
**Q9Y6M5**	Zinc transporter 1	0.71
**P11717**	Cation-independent mannose-6-phosphate receptor	0.72
**P21796**	Voltage-dependent anion-selective channel protein 1	0.72
**Q15365**	Poly(rC)-binding protein 1	0.72
**Q15738**	Sterol-4-alpha-carboxylate 3-dehydrogenase, decarboxylating	0.72
**Q969H8**	UPF0556 protein C19orf10	0.73
**P04406**	Glyceraldehyde-3-phosphate dehydrogenase	0.73
**P49327**	Fatty acid synthase	0.74
**Q9H3N1**	Thioredoxin-related transmembrane protein 1	0.74
**P27797**	Calreticulin	0.74
**P38646**	Stress-70 protein, mitochondrial	0.74
**P62805**	Histone H4	0.75

**P04083**	Annexin A1	0.75
**Q9Y490**	Talin-1	0.75
**P46940**	Ras GTPase-activating-like protein IQGAP1	0.75
**P23284**	Peptidyl-prolyl cis-trans isomerase B	0.76
**Q15907**	Ras-related protein Rab-11B	0.76
**P05556**	Integrin beta-1	0.77
**Q06830**	Peroxiredoxin-1	0.77
**O95573**	Long-chain-fatty-acid--CoA ligase 3	0.78
**P00505**	Aspartate aminotransferase, mitochondrial	0.78
**P30043**	Flavin reductase (NADPH)	0.78
**P43007**	Neutral amino acid transporter A	0.79
**P55809**	Succinyl-CoA:3-ketoacid coenzyme A transferase 1, mitochondrial	0.79
**Q14764**	Major vault protein	0.79
**P54920**	Alpha-soluble NSF attachment protein	0.81
**Q13011**	Delta(3,5)-Delta(2,4)-dienoyl-CoA isomerase, mitochondrial	0.81
**P23458**	Tyrosine-protein kinase JAK1	0.82
**P12277**	Creatine kinase B-type	0.82
**Q9Y5M8**	Signal recognition particle receptor subunit beta	0.82
**P01891**	HLA class I histocompatibility antigen, A-68 alpha chain	0.82
**Q9UBS4**	DnaJ homolog subfamily B member 11	0.83
**O96008**	Mitochondrial import receptor subunit TOM40 homolog	0.83
**P07195**	L-lactate dehydrogenase B chain	0.84
**P84095**	Rho-related GTP-binding protein RhoG	0.84
**P68371**	Tubulin beta-4B chain	0.86
**O15173**	Membrane-associated progesterone receptor component 2	0.86
**Q96QK1**	Vacuolar protein sorting-associated protein 35	0.87
**Q01581**	Hydroxymethylglutaryl-CoA synthase, cytoplasmic	0.87
**B4DXP9**	Alpha-centractin	0.88
**P55072**	Transitional endoplasmic reticulum ATPase	0.88
**P61026**	Ras-related protein Rab-10	0.91
**P60842**	Eukaryotic initiation factor 4A-I	0.92

**Q9UQ80**	Proliferation-associated protein 2G4	0.93
**Q9Y678**	Coatomer subunit gamma-1	0.93
**Q9H853**	Putative tubulin-like protein alpha-4B	0.94
**Q9Y696**	Chloride intracellular channel protein 4	0.95
**Q02790**	Peptidyl-prolyl cis-trans isomerase FKBP4	0.96
**Q8TEM1**	Nuclear pore membrane glycoprotein 210	0.96
**Q13162**	Peroxiredoxin-4	0.97
**Q9Y617**	Phosphoserine aminotransferase	1.00
**P56199**	Integrin alpha-1	1.00
**P04843**	Dolichyl-diphosphooligosaccharide--protein glycosyltransferase subunit 1	1.02
**P06733**	Alpha-enolase	1.02
**P07737**	Profilin-1	1.02
**P04792**	Heat shock protein beta-1	1.02
**P35579**	Myosin-9	1.03
**Q92616**	Translational activator GCN1	1.03
**O75396**	Vesicle-trafficking protein SEC22b	1.04
**Q04917**	14-3-3 protein eta	1.05
**Q8NF37**	Lysophosphatidylcholine acyltransferase 1	1.06
**Q9UBF2**	Coatomer subunit gamma-2	1.10
**P11169**	Solute carrier family 2, facilitated glucose transporter member 3	1.12
**Q9UL25**	Ras-related protein Rab-21	1.14
**P61981**	14-3-3 protein gamma	1.14
**Q15758**	Neutral amino acid transporter B(0)	1.16
**Q15181**	Inorganic pyrophosphatase	1.17
**P18669**	Phosphoglycerate mutase 1	1.18
**Q9UQE7**	Structural maintenance of chromosomes protein 3	1.28
**P11021**	78 kDa glucose-regulated protein	1.29
**P05141**	ADP/ATP translocase 2	1.33
**P05362**	Intercellular adhesion molecule 1	1.33
**P05161**	Ubiquitin-like protein ISG15	1.37
**P12236**	ADP/ATP translocase 3	1.38

**P41250**	Glycine--tRNA ligase	1.48
**P49588**	Alanine--tRNA ligase, cytoplasmic	1.55
**P35232**	Prohibitin	1.62
**O43175**	D-3-phosphoglycerate dehydrogenase	1.74
**P49411**	Elongation factor Tu, mitochondrial	1.82
**P42704**	Leucine-rich PPR motif-containing protein, mitochondrial	2.15
**P10809**	60 kDa heat shock protein, mitochondrial	2.73

*：Heavy/Light means the protein expression ratio of PI4KIIα RNAi MCF-7 cells to Control cells.

Table S2. Protein targets identified by SILAC and LC-MS/MS for control MCF-7 cells (sample 3) and combined inhibition MCF-7 cells (sample 4).

Accession	Description	Heavy/Light*
**Q09666**	Neuroblast differentiation-associated protein AHNAK	0.21
**Q15459**	Splicing factor 3A subunit 1	0.28
**P16401**	Histone H1.5	0.29
**Q92945**	Far upstream element-binding protein 2	0.34
**Q9UNX4**	WD repeat-containing protein 3	0.34
**Q96PK6**	RNA-binding protein 14	0.36
**O94776**	Metastasis-associated protein MTA2	0.38
**Q05048**	Cleavage stimulation factor subunit 1	0.41
**O15213**	WD repeat-containing protein 46	0.43
**P14866**	Heterogeneous nuclear ribonucleoprotein L	0.43
**Q8NI36**	WD repeat-containing protein 36	0.44
**H7C267**	Pescadillo homolog (Fragment)	0.45
**Q15393**	Splicing factor 3B subunit 3	0.45
**P08670**	Vimentin	0.46
**P46063**	ATP-dependent DNA helicase Q1	0.46
**Q12996**	Cleavage stimulation factor subunit 3	0.46
**Q9BV38**	WD repeat-containing protein 18	0.46
**P15924**	Desmoplakin	0.47
**F6XZQ7**	Glutathione S-transferase Mu 2	0.48
**P26599**	Polypyrimidine tract-binding protein 1	0.48
**P11387**	DNA topoisomerase 1	0.48
**P09874**	Poly [ADP-ribose] polymerase 1	0.48
**Q01082**	Spectrin beta chain, non-erythrocytic 1	0.48
**P49327**	Fatty acid synthase	0.49
**P62750**	60S ribosomal protein L23a	0.50
**P08579**	U2 small nuclear ribonucleoprotein B	0.51
**P04792**	Heat shock protein beta-1	0.51

**P12270**	Nucleoprotein TPR	0.51
**Q2TAY7**	WD40 repeat-containing protein SMU1	0.51
**Q15050**	Ribosome biogenesis regulatory protein homolog	0.52
**P12277**	Creatine kinase B-type	0.53
**P32119**	Peroxiredoxin-2	0.54
**P23246**	Splicing factor, proline- and glutamine-rich	0.54
**Q9BUQ8**	Probable ATP-dependent RNA helicase DDX23	0.54
**P17066**	Heat shock 70 kDa protein 6	0.55
**Q92499**	ATP-dependent RNA helicase DDX1	0.55
**Q9P258**	Protein RCC2	0.56
**Q9Y224**	UPF0568 protein C14orf166	0.56
**P13010**	X-ray repair cross-complementing protein 5	0.56
**P49736**	DNA replication licensing factor MCM2	0.56
**P80723**	Brain acid soluble protein 1	0.56
**P38919**	Eukaryotic initiation factor 4A-III	0.57
**Q12905**	Interleukin enhancer-binding factor 2	0.57
**Q9NS69**	Mitochondrial import receptor subunit TOM22 homolog	0.57
**Q15269**	Periodic tryptophan protein 2 homolog	0.58
**O75533**	Splicing factor 3B subunit 1	0.58
**Q13907-2**	Isoform 2 of Isopentenyl-diphosphate Delta-isomerase 1	0.59
**Q08945**	FACT complex subunit SSRP1	0.59
**Q9GZL7**	Ribosome biogenesis protein WDR12	0.59
**O60264**	SWI/SNF-related matrix-associated actin-dependent regulator of chromatin subfamily A member 5	0.602
**P62304**	Small nuclear ribonucleoprotein E	0.60
**Q15397**	Pumilio domain-containing protein KIAA0020	0.61
**Q8IY81**	pre-rRNA processing protein FTSJ3	0.61
**O43809**	Cleavage and polyadenylation specificity factor subunit 5	0.61
**P52907**	F-actin-capping protein subunit alpha-1	0.61
**Q00839**	Heterogeneous nuclear ribonucleoprotein U	0.61
**Q08211**	ATP-dependent RNA helicase A	0.62

**Q7KZF4**	Staphylococcal nuclease domain-containing protein 1	0.62
**Q9Y3T9**	Nucleolar complex protein 2 homolog	0.63
**Q14683**	Structural maintenance of chromosomes protein 1A	0.63
**Q05639**	Elongation factor 1-alpha 2	0.64
**O75691**	Small subunit processome component 20 homolog	0.64
**Q8IWA0**	WD repeat-containing protein 75	0.64
**Q9Y678**	Coatomer subunit gamma-1	0.64
**Q13151**	Heterogeneous nuclear ribonucleoprotein A0	0.65
**O15144**	Actin-related protein 2/3 complex subunit 2	0.65
**P15121**	Aldose reductase	0.65
**Q96GQ7**	Probable ATP-dependent RNA helicase DDX27	0.65
**P52292**	Importin subunit alpha-2	0.66
**P56182**	Ribosomal RNA processing protein 1 homolog A	0.66
**Q12788**	Transducin beta-like protein 3	0.66
**P23284**	Peptidyl-prolyl cis-trans isomerase B	0.66
**O75531**	Barrier-to-autointegration factor	0.67
**Q9Y2P8**	RNA 3'-terminal phosphate cyclase-like protein	0.67
**Q9NVP1**	ATP-dependent RNA helicase DDX18	0.67
**P16083**	Ribosyldihydronicotinamide dehydrogenase [quinone]	0.68
**P20700**	Lamin-B1	0.68
**Q9Y5B9**	FACT complex subunit SPT16	0.68
**Q06203**	Amidophosphoribosyltransferase	0.69
**O00148**	ATP-dependent RNA helicase DDX39A	0.69
**O43818**	U3 small nucleolar RNA-interacting protein 2	0.69
**Q03701**	CCAAT/enhancer-binding protein zeta	0.70
**Q6P2Q9**	Pre-mRNA-processing-splicing factor 8	0.70
**P37802**	Transgelin-2	0.71
**P30740**	Leukocyte elastase inhibitor	0.71
**P08574**	Cytochrome c1, heme protein, mitochondrial	0.72
**Q9UQE7**	Structural maintenance of chromosomes protein 3	0.72
**Q9Y2W1**	Thyroid hormone receptor-associated protein 3	0.72

**Q14690**	Protein RRP5 homolog	0.72
**P53004**	Biliverdin reductase A	0.72
**Q15365**	Poly(rC)-binding protein 1	0.72
**Q9Y5J1**	U3 small nucleolar RNA-associated protein 18 homolog	0.73
**P35237**	Serpin B6	0.74
**P36871**	Phosphoglucomutase-1	0.74
**Q8TDN6**	Ribosome biogenesis protein BRX1 homolog	0.74
**O00232**	26S proteasome non-ATPase regulatory subunit 12	0.74
**P08238**	Heat shock protein HSP 90-beta	0.75
**E9PBF6**	Lamin-B1	0.75
**Q15738**	Sterol-4-alpha-carboxylate 3-dehydrogenase, decarboxylating	0.75
**O60812**	Heterogeneous nuclear ribonucleoprotein C-like 1	0.75
**P05783**	Keratin, type I cytoskeletal 18	0.76
**O00567**	Nucleolar protein 56	0.77
**P04080**	Cystatin-B	0.77
**Q9Y295**	Developmentally-regulated GTP-binding protein 1	0.78
**Q92979**	Ribosomal RNA small subunit methyltransferase NEP1	0.78
**P31949**	Protein S100-A11	0.78
**Q96DG6**	Carboxymethylenebutenolidase homolog	0.78
**Q9NV31**	U3 small nucleolar ribonucleoprotein protein IMP3	0.78
**P05386**	60S acidic ribosomal protein P1	0.78
**P09382**	Galectin-1	0.78
**Q86U38**	Nucleolar protein 9	0.78
**P25815**	Protein S100-P	0.79
**Q02790**	Peptidyl-prolyl cis-trans isomerase FKBP4	0.79
**Q9BYZ2**	L-lactate dehydrogenase A-like 6B	0.79
**P49207**	60S ribosomal protein L34	0.79
**Q9NYH9**	U3 small nucleolar RNA-associated protein 6 homolog	0.79
**P22314**	Ubiquitin-like modifier-activating enzyme 1	0.81
**P26038**	Moesin	0.81
**P26447**	Protein S100-A4	0.81

**O43143**	Putative pre-mRNA-splicing factor ATP-dependent RNA helicase DHX15	0.81
**P48047**	ATP synthase subunit O, mitochondrial	0.82
**P29317**	Ephrin type-A receptor 2	0.82
**P31947**	14-3-3 protein sigma	0.82
**P30086**	Phosphatidylethanolamine-binding protein 1	0.82
**P34932**	Heat shock 70 kDa protein 4	0.83
**P58107**	Epiplakin	0.83
**Q9BZK3**	Putative nascent polypeptide-associated complex subunit alpha-like protein	0.83
**Q9Y2X3**	Nucleolar protein 58	0.83
**O60287**	Nucleolar pre-ribosomal-associated protein 1	0.83
**F5H0T1**	Stress-induced-phosphoprotein 1	0.83
**P28066**	Proteasome subunit alpha type-5	0.84
**P28072**	Proteasome subunit beta type-6	0.84
**P35232**	Prohibitin	0.84
**P62424**	60S ribosomal protein L7a	0.84
**P49721**	Proteasome subunit beta type-2	0.85
**P62854**	40S ribosomal protein S26	0.86
**P18669**	Phosphoglycerate mutase 1	0.86
**P00491**	Purine nucleoside phosphorylase	0.86
**P26373**	60S ribosomal protein L13	0.86
**P07195**	L-lactate dehydrogenase B chain	0.87
**P21796**	Voltage-dependent anion-selective channel protein 1	0.87
**Q01581**	Hydroxymethylglutaryl-CoA synthase, cytoplasmic	0.87
**Q92621**	Nuclear pore complex protein Nup205	0.88
**P62081**	40S ribosomal protein S7	0.88
**Q06830**	Peroxiredoxin-1	0.88
**E9PCK7**	RRP12-like protein	0.89
**Q99436**	Proteasome subunit beta type-7	0.89
**Q99497**	Protein DJ-1	0.89
**P31153**	S-adenosylmethionine synthase isoform type-2	0.89
**P06744**	Glucose-6-phosphate isomerase	0.90

**Q9UL46**	Proteasome activator complex subunit 2	0.90
**P20618**	Proteasome subunit beta type-1	0.90
**P30041**	Peroxiredoxin-6	0.90
**P30050**	60S ribosomal protein L12	0.90
**P78527**	DNA-dependent protein kinase catalytic subunit	0.90
**Q9NV06**	DDB1- and CUL4-associated factor 13	0.90
**Q9Y230**	RuvB-like 2	0.90
**P06703**	Protein S100-A6	0.91
**P25705**	ATP synthase subunit alpha, mitochondrial	0.91
**P49257**	Protein ERGIC-53	0.91
**A6NDG6**	Phosphoglycolate phosphatase	0.91
**P61586**	Transforming protein RhoA	0.91
**P62263**	40S ribosomal protein S14	0.91
**Q562R1**	Beta-actin-like protein 2	0.91
**P23921**	Ribonucleoside-diphosphate reductase large subunit	0.92
**P51571**	Translocon-associated protein subunit delta	0.92
**P04406**	Glyceraldehyde-3-phosphate dehydrogenase	0.92
**P42166**	Lamina-associated polypeptide 2, isoform alpha	0.92
**P62277**	40S ribosomal protein S13	0.92
**P06733**	Alpha-enolase	0.92
**P42766**	60S ribosomal protein L35	0.92
**O00299**	Chloride intracellular channel protein 1	0.92
**O95336**	6-phosphogluconolactonase	0.92
**P07737**	Profilin-1	0.92
**P13639**	Elongation factor 2	0.92
**O75131**	Copine-3	0.93
**P08754**	Guanine nucleotide-binding protein G(k) subunit alpha	0.93
**P47914**	60S ribosomal protein L29	0.93
**P08708**	40S ribosomal protein S17	0.93
**P25787**	Proteasome subunit alpha type-2	0.93
**P30084**	Enoyl-CoA hydratase, mitochondrial	0.93

**P0CG39**	POTE ankyrin domain family member J	0.94
**P35268**	60S ribosomal protein L22	0.94
**P41091**	Eukaryotic translation initiation factor 2 subunit 3	0.94
**Q01813**	6-phosphofructokinase type C	0.94
**Q8N726**	Cyclin-dependent kinase inhibitor 2A, isoform 4	0.94
**P18124**	60S ribosomal protein L7	0.94
**P25398**	40S ribosomal protein S12	0.94
**Q9H9B4**	Sideroflexin-1	0.94
**P46940**	Ras GTPase-activating-like protein IQGAP1	0.95
**Q9BQ39**	ATP-dependent RNA helicase DDX50	0.95
**P24534**	Elongation factor 1-beta	0.95
**P12004**	Proliferating cell nuclear antigen	0.96
**Q16719**	Kynureninase	0.96
**P52597**	Heterogeneous nuclear ribonucleoprotein F	0.96
**P62269**	40S ribosomal protein S18	0.96
**P61254**	60S ribosomal protein L26	0.96
**Q14019**	Coactosin-like protein	0.96
**F5H1L6**	Serine/threonine-protein phosphatase PP1-alpha catalytic subunit (Fragment)	0.97
**P16152**	Carbonyl reductase [NADPH] 1	0.97
**P46777**	60S ribosomal protein L5	0.97
**Q15181**	Inorganic pyrophosphatase	0.97
**P61313**	60S ribosomal protein L15	0.98
**Q9HAV0**	Guanine nucleotide-binding protein subunit beta-4	0.99
**P51153**	Ras-related protein Rab-13	0.99
**O75489**	NADH dehydrogenase [ubiquinone] iron-sulfur protein 3, mitochondrial	0.99
**I3L4N8**	Actin, cytoplasmic 2 (Fragment)	0.99
**P00367**	Glutamate dehydrogenase 1, mitochondrial	0.99
**Q9Y3U8**	60S ribosomal protein L36	0.99
**P62249**	40S ribosomal protein S16	0.99
**E7ES52**	Cyclin-dependent kinase inhibitor 1B	1.00
**P21266**	Glutathione S-transferase Mu 3	1.00

**P60903**	Protein S100-A10	1.00
**Q96AG4**	Leucine-rich repeat-containing protein 59	1.00
**Q8TD16-2**	Isoform 2 of Protein bicaudal D homolog 2	1.00
**P00441**	Superoxide dismutase [Cu-Zn]	1.01
**P62280**	40S ribosomal protein S11	1.01
**P62266**	40S ribosomal protein S23	1.01
**Q9UBI6**	Guanine nucleotide-binding protein G(I)/G(S)/G(O) subunit gamma-12	1.01
**P46782**	40S ribosomal protein S5	1.02
**P55072**	Transitional endoplasmic reticulum ATPase	1.02
**P15311**	Ezrin	1.02
**P53985**	Monocarboxylate transporter 1	1.02
**P53999**	Activated RNA polymerase II transcriptional coactivator p15	1.02
**P62701**	40S ribosomal protein S4, X isoform	1.02
**P23458**	Tyrosine-protein kinase JAK1	1.03
**Q92616**	Translational activator GCN1	1.03
**P61353**	60S ribosomal protein L27	1.03
**P62906**	60S ribosomal protein L10a	1.03
**P31689**	DnaJ homolog subfamily A member 1	1.03
**P27348**	14-3-3 protein theta	1.04
**P30041**	Peroxiredoxin-6	1.04
**P23526**	Adenosylhomocysteinase	1.04
**P50914**	60S ribosomal protein L14	1.04
**P14625**	Endoplasmin	1.04
**P62841**	40S ribosomal protein S15	1.04
**Q9NR45**	Sialic acid synthase	1.04
**Q969H8**	UPF0556 protein C19orf10	1.05
**P28070**	Proteasome subunit beta type-4	1.05
**P43121**	Cell surface glycoprotein MUC18	1.05
**Q969Q0**	60S ribosomal protein L36a-like	1.05
**P35579**	Myosin-9	1.05
**O76003**	Glutaredoxin-3	1.06

**P55795**	Heterogeneous nuclear ribonucleoprotein H2	1.06
**P61006**	Ras-related protein Rab-8A	1.07
**O00487**	26S proteasome non-ATPase regulatory subunit 14	1.07
**O43707**	Alpha-actinin-4	1.07
**Q99714**	3-hydroxyacyl-CoA dehydrogenase type-2	1.08
**O75643**	U5 small nuclear ribonucleoprotein 200 kDa helicase	1.08
**P46778**	60S ribosomal protein L21	1.08
**Q04917**	14-3-3 protein eta	1.08
**P40429**	60S ribosomal protein L13a	1.09
**Q12907**	Vesicular integral-membrane protein VIP36	1.09
**Q15006**	ER membrane protein complex subunit 2	1.09
**P68371**	Tubulin beta-4B chain	1.10
**Q9H3N1**	Thioredoxin-related transmembrane protein 1	1.10
**H0YKF0**	Electron transfer flavoprotein subunit alpha, mitochondrial (Fragment)	1.11
**P37837**	Transaldolase	1.11
**P07814**	Bifunctional glutamate/proline--tRNA ligase	1.11
**P05198**	Eukaryotic translation initiation factor 2 subunit 1	1.11
**Q01105**	Protein SET	1.12
**P62805**	Histone H4	1.12
**Q9UMS4**	Pre-mRNA-processing factor 19	1.12
**P05556**	Integrin beta-1	1.12
**P22087**	rRNA 2'-O-methyltransferase fibrillarin	1.13
**Q16795**	NADH dehydrogenase [ubiquinone] 1 alpha subcomplex subunit 9, mitochondrial	1.14
**P19338**	Nucleolin	1.14
**Q9H4A6**	Golgi phosphoprotein 3	1.14
**P04083**	Annexin A1	1.15
**Q9Y5M8**	Signal recognition particle receptor subunit beta	1.15
**P55809**	Succinyl-CoA:3-ketoacid coenzyme A transferase 1, mitochondrial	1.15
**P84243**	Histone H3.3	1.15
**Q13641**	Trophoblast glycoprotein	1.16
**P11166**	Solute carrier family 2, facilitated glucose transporter member 1	1.16

**P02792**	Ferritin light chain	1.16
**Q14764**	Major vault protein	1.17
**P48637**	Glutathione synthetase	1.17
**P30040**	Endoplasmic reticulum resident protein 29	1.188
**Q10589**	Bone marrow stromal antigen 2	1.19
**O96008**	Mitochondrial import receptor subunit TOM40 homolog	1.19
**P31930**	Cytochrome b-c1 complex subunit 1, mitochondrial	1.19
**O75874**	Isocitrate dehydrogenase [NADP] cytoplasmic	1.19
**P54920**	Alpha-soluble NSF attachment protein	1.19
**Q71DI3**	Histone H3.2	1.20
**P00505**	Aspartate aminotransferase, mitochondrial	1.20
**P60842**	Eukaryotic initiation factor 4A-I	1.21
**P50148**	Guanine nucleotide-binding protein G(q) subunit alpha	1.22
**P00966**	Argininosuccinate synthase	1.22
**P04843**	Dolichyl-diphosphooligosaccharide--protein glycosyltransferase subunit 1	1.23
**Q96QK1**	Vacuolar protein sorting-associated protein 35	1.24
**O14980**	Exportin-1	1.24
**Q9BVK6**	Transmembrane emp24 domain-containing protein 9	1.24
**Q13011**	Delta(3,5)-Delta(2,4)-dienoyl-CoA isomerase, mitochondrial	1.25
**P27797**	Calreticulin	1.26
**Q01650**	Large neutral amino acids transporter small subunit 1	1.26
**H7BZJ3**	Thioredoxin (Fragment)	1.28
**P12236**	ADP/ATP translocase 3	1.28
**P00492**	Hypoxanthine-guanine phosphoribosyltransferase	1.29
**P30043**	Flavin reductase (NADPH)	1.29
**O95373**	Importin-7	1.31
**O60568**	Procollagen-lysine,2-oxoglutarate 5-dioxygenase 3	1.32
**P62745**	Rho-related GTP-binding protein RhoB	1.34
**Q07065**	Cytoskeleton-associated protein 4	1.34
**P10599**	Thioredoxin	1.35
**P40261**	Nicotinamide N-methyltransferase	1.35

**Q16740**	Putative ATP-dependent Clp protease proteolytic subunit, mitochondrial	1.36
**O95573**	Long-chain-fatty-acid--CoA ligase 3	1.37
**Q96HE7**	ERO1-like protein alpha	1.37
**P48556**	26S proteasome non-ATPase regulatory subunit 8	1.38
**P01891**	HLA class I histocompatibility antigen, A-68 alpha chain	1.38
**P61981**	14-3-3 protein gamma	1.39
**P67936**	Tropomyosin alpha-4 chain	1.40
**P61106**	Ras-related protein Rab-14	1.41
**O43681**	ATPase ASNA1	1.42
**O75396**	Vesicle-trafficking protein SEC22b	1.42
**H7C3T4**	Peroxiredoxin-4 (Fragment)	1.44
**P05141**	ADP/ATP translocase 2	1.44
**Q04837**	Single-stranded DNA-binding protein, mitochondrial	1.47
**Q9UHV9**	Prefoldin subunit 2	1.53
**P13667**	Protein disulfide-isomerase A4	1.55
**Q6UB35**	Monofunctional C1-tetrahydrofolate synthase, mitochondrial	1.60
**Q8NFH3**	Nucleoporin Nup43	1.60
**Q15645**	Pachytene checkpoint protein 2 homolog	1.62
**Q8IV08**	Phospholipase D3	1.62
**P24752**	Acetyl-CoA acetyltransferase, mitochondrial	1.64
**P05161**	Ubiquitin-like protein ISG15	1.66
**Q14566**	DNA replication licensing factor MCM6	1.71
**P05362**	Intercellular adhesion molecule 1	1.72
**O15173**	Membrane-associated progesterone receptor component 2	1.78
**Q8TEM1**	Nuclear pore membrane glycoprotein 210	1.81
**P21266**	Glutathione S-transferase Mu 3	1.84
**P61026**	Ras-related protein Rab-10	1.87
**P49755**	Transmembrane emp24 domain-containing protein 10	1.88
**P11021**	78 kDa glucose-regulated protein	1.97
**P49411**	Elongation factor Tu, mitochondrial	2.00
**P41250**	Glycine--tRNA ligase	2.17

**P42704**	Leucine-rich PPR motif-containing protein, mitochondrial	2.27
**Q15758**	Neutral amino acid transporter B(0)	2.32
**P24539**	ATP synthase subunit b, mitochondrial	2.48
**P40939**	Trifunctional enzyme subunit alpha, mitochondrial	2.50
**Q07021**	Complement component 1 Q subcomponent-binding protein, mitochondrial	2.52
**O43175**	D-3-phosphoglycerate dehydrogenase	2.52=3
**P10809**	60 kDa heat shock protein, mitochondrial	2.67
**Q9Y617**	Phosphoserine aminotransferase	2.87]
**P49588**	Alanine--tRNA ligase, cytoplasmic	2.88]
**Q14204**	Cytoplasmic dynein 1 heavy chain 1	3.04]
**P38646**	Stress-70 protein, mitochondrial	3.48]
**E9PPU0**	Epiplakin	4.29

*：Heavy/Light means the protein expression ratio of PI4KIIα and EGFR dual inhibited MCF-7 cells to Control cells.
